# Supplementary figures and images for: Evaluation of the New SingularityTM Air versus Ambu® Aura GainTM: A Randomized, Crossover Mannequin Study
Source: J Clin Med. 2022 Dec 7;11(24):7266. doi: 10.3390/jcm11247266 (PMC9787694; doi:10.3390/jcm11247266)

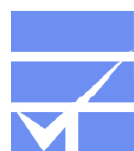

# CONSORT

TRANSPARENT REPORTING of TRIALS

## CONSORT 2010 Flow Diagram

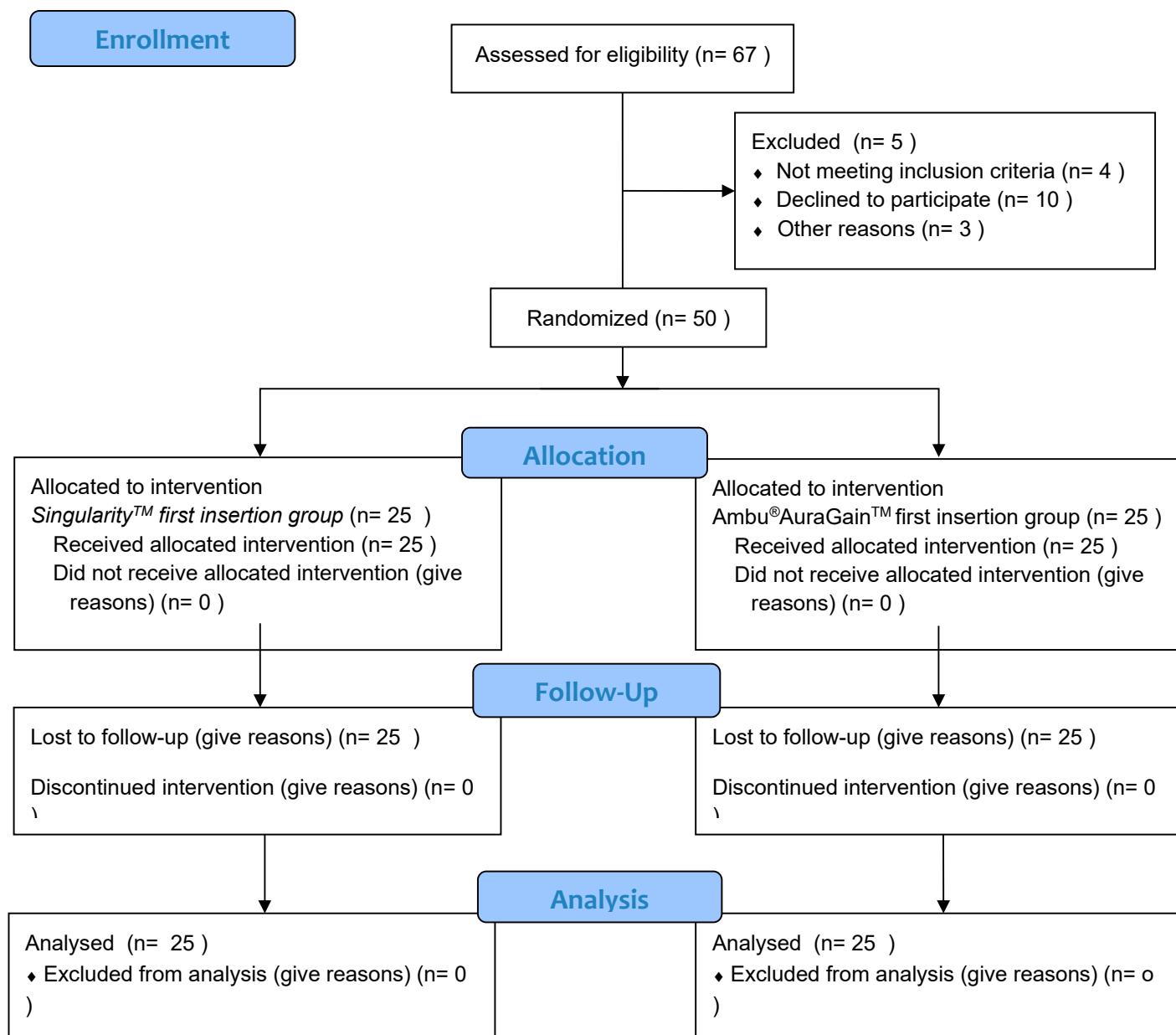

Supplement: Supplementary file 1 [file jcm-11-07266-s001.zip › jcm-2035325-supplementary.pdf]
